# Regenerative Medicine Advancements: A Systematic Review on the Combinatory Effect of Platelet‐Rich Plasma/Fibrin and Collagen

**DOI:** 10.1155/ijbm/1679626

**Published:** 2026-01-12

**Authors:** Nunzia Gallo, Chiara Kodra, Domenico Rocco, Cosimo Saponaro, Alessandro Sannino, Luca Salvatore

**Affiliations:** ^1^ Typeone Biomaterials S.r.l., Calimera, Italy; ^2^ Department of Engineering for Innovation, University of Salento, Lecce, Italy, unisalento.it; ^3^ Division of Heart Surgery, “V. Fazzi” Hospital, Lecce, Italy; ^4^ Department of Experimental Medicine, University of Salento, Lecce, Italy, unisalento.it

**Keywords:** collagen, platelet-rich fibrin, platelet-rich plasma, tissues regeneration

## Abstract

**Background:**

The regeneration of injured tissues remains a major clinical challenge. Among emerging biomaterials, collagen with platelet‐rich plasma (PRP) or platelet‐rich fibrin (PRF) showed promising outcomes, individually and in combination.

**Objective:**

To systematically review clinical evidence on the efficacy, applications, and safety of PRP/PRF and collagen for regenerative medicine applications.

**Methods:**

A systematic literature search was conducted in PubMed, Wiley Online Library, Google Scholar, and ClinicalTrials.gov (search date: September, 2025). Inclusion criteria: clinical studies evaluating PRP/PRF and collagen formulations. Exclusion: preclinical only or nonoriginal research. Data were synthesized narratively.

**Results:**

Twenty‐six clinical studies were included. Applications included gingival recession, periodontitis, tendon injuries, bone regeneration, peripheral nerve repair, and chronic ulcers. Most studies reported positive outcomes, though many lacked control groups or had small sample sizes. No serious adverse events were reported.

**Conclusion:**

PRP/PRF and collagen show potential for various clinical applications in regenerative medicine. However, randomized clinical studies are necessary to demonstrate their superiority to standard treatment and to standardize protocols.

## 1. Introduction

Truly effective strategies that allow complete regeneration of body lesions of any type and the restoration of the physiological functioning of organs are very few. Despite decades of preclinical and clinical studies and the myriads of compounds tested, achieving a high regenerative rate is still an open and challenging dare. The search for cost‐effective, easy‐to‐use, one‐step, and ready‐to‐use strategies is even in turmoil. Therefore, the search for new bioactive materials is incessant. However, nowadays almost all strategies and materials potentials have been revealed. A method that allows increasing the bioactivity of the materials consists in their combination. In this respect, biomaterials that are particularly relevant for their extraordinary clinical efficacy are blood derivatives and collagen.

Among blood derivatives, platelet‐rich plasma (PRP) and platelet‐rich fibrin (PRF) are the most studied and used since the 1980 due to their regenerative potential and autologous origin [1–4]. PRP typically presents as a low‐density fibrin network in an injectable gel form, whereas PRF is a high‐density, stress‐resistant gel, noninjectable. PRP is isolated from anticoagulated blood. A high variability exists in their isolation protocols that differ also in harvesting systems (special tubes, syringe systems, automated devices, centrifugation bags) [5]. The most widely used method for PRP/PRF preparation is by means of centrifugation, but different protocols are applied with different speed, time, volume, and number of separation steps. Usually, PRF isolation protocols consist of a single centrifugation step, while PRP isolation protocols are based on a double centrifugation [1]. Briefly, as regards PRP, after the blood clotting cascade block by an anticoagulant‐containing solution (usually sodium citrate or ACD‐A), a first centrifugation step allows us to separate red and white blood cells from plasma and platelets. Then, a second centrifugation is performed to concentrate PRP. Once isolated, PRP could be directly applied or activated by means of chemical (e.g., collagen, thrombin, thrombin receptor agonist peptide, adenosine diphosphate, Ca^2+^) or physical (e.g., pulse‐electric field, light) methods to induce growth factors release [5].

PRF preparations are highly variable and often poorly reported, besides consisting of one centrifugation step. Unlike PRP, PRF is isolated from patients’ blood collected without the addition of anticoagulants. After immediate centrifugation of freshly harvested blood, coagulation naturally occurs, yielding a dense fibrin clot enriched in platelets and leukocytes. While PRF protocols are relatively more uniform, device and spin parameters can still affect blend and performance [6].

The reproducibility of production methods is hindered by several missing information (e.g., PRP/PRF volume or platelet concentration) [4]. Additionally, patient‐related factors (age, sex, clinical condition, medication, hematocrit) affect its bioactivity, hindering its real efficacy [5]. This lack of standardization yields PRP/PRF products with different cellular and protein compositions (0.3 × 10^6^ − 2.3 × 10^6^ platelet/μL) [5] and, consequently, different biological activities, complicating comparisons between studies and reproducibility.

Additionally, the variability in PRP/PRF composition is not only related to differences in platelet concentration but also to the inclusion or exclusion of leukocytes. This aspect represents one of the most debated issues in the field of regenerative medicine, as leukocytes can profoundly affect the biological behavior of PRP/PRF. On one hand, leukocyte‐rich (LR) concentrates may provide additional growth factors, cytokines, and immune mediators that can promote antimicrobial activity and stimulate tissue regeneration. On the other hand, the presence of leukocytes, particularly neutrophils, has been associated with the release of proinflammatory cytokines and proteolytic enzymes, which may exacerbate inflammation and impair healing in certain contexts. LR concentrates were found to provide pain relief and successful outcomes for patients with lateral epicondylitis compared with alternative leukocyte‐poor (LP) concentrate injections [7]. Similarly, LR formulations were found to reach higher wound healing rates than the LP formulations, with a more prominent angiogenesis and regulated inflammatory response [8]. Conversely, in the case of articular cartilage lesions, PRP without concentrated leukocytes was found to be more suitable [9].

Consequently, the inclusion or exclusion of leukocytes significantly alters the biochemical and clinical profile of blood derivative formulations, leading to inconsistent outcomes across studies with a strict dependence for the clinical application. This heterogeneity complicates the interpretation and comparison of published results, underlining the need for standardized classification systems and detailed reporting of PRP/PRF preparation protocols to ensure reproducibility and allow meaningful clinical correlations [6].

Platelet concentrates enhance healing and regenerative processes because of their richness in bioactive factors such as growth factors (e.g., transforming growth factor‐β, platelet‐derived growth factor, insulin‐like growth factor I and II, fibroblast growth factor, epidermal growth factor, vascular endothelial growth factor, endothelial cell growth factor), clotting factors, proteases, antiproteases, as well as other compounds such as serotonin, histamine, dopamine, and catecholamine [1]. Indeed, their application in the treatment of specific issues of several body apparatus is well documented. Among them, the most well known belongs to the integumentary (e.g., wrinkles, acne scars, severe wounds, hair) [2, 10–12], the musculoskeletal (e.g., epicondylitis, tendinopathies, ligament tears, osteoarthritis, cartilage defects, fasciitis) [12, 13], and the urinary (e.g., incontinence, fistulas, prolapse, atrophy) [14] systems.

However, the drawbacks of blood derivatives (e.g., low half‐life, low mechanical properties) have driven their combination with other biomaterials to improve efficacy. Some attempts were made to mix PRP/PRF with biomaterials like type I collagen [15–20] or hyaluronic acid, [21, 22], with positive in vitro, preclinical, and clinical outcomes. Although traceable and concluded studies are very few, blends of PRP and collagen (PRP/Coll) or of PRF and collagen (PRF/Coll) were found to be particularly promising thanks to the intrinsic scaffolding and bioactive properties of collagen. Type I collagen is the most important structural protein of vertebrates, accounting for about 30% of the total protein weight of the human body [23]. Being one of the most preserved extracellular matrix components, it plays fundamental structural and nonstructural roles that are particularly attractive for biomedical applications [24]. Indeed, type I collagen has been recognized as the gold standard material and has been used as biomaterial for the development of pro‐regenerative medical devices since 1970 [25]. Applicative areas of collagen‐based devices are thus endless. Wound healing, antiaging, and osteochondral regeneration are well‐established quite common clinical practices [23, 26].

**Figure 1:**
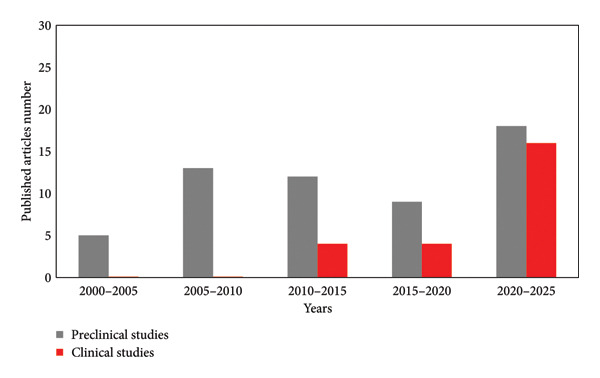
Number of published preclinical and clinical studies on blends of platelet concentrates (PRP or PRF) and collagen, from 2000 to 2025.

Thus, the combination of a pro‐regenerative protein that has been elected as a gold standard biomaterial with growth factors‐rich blood extracts could afford to reach superior effectiveness. However, while PRP/PRF has been clinically used since the 1980s [2] and collagen since the 1970s, the combinatory effect of PRP/PRF with collagen started to be investigated only recently. Some in vitro [18, 20, 27] and preclinical [17, 19, 28–37] studies have been performed on PRP/PRF and collagen blends, but few clinical studies [15, 16, 38–50] have been reported. To the best of our knowledge, these blends began to be clinically evaluated only in 2009. However, the number of published articles suggests how this topic is of great interest since research is constantly going on preclinical models, and even more clinical studies have been approved by ethical committees (Figure 1). Indeed, after the publication of the results of the first five clinical studies, 4 works were published between 2015 and 2020, and 17 between 2020 and 2024 (Table S1), suggesting the increasing interest in such materials combinations. Blood derivatives and collagen blends were found to have been mainly used for the treatment of gingival recessions, periodontitis, ulcers, tendinopathies, and bone regeneration. Among them, particularly relevant were results obtained for the treatment of periodontitis.

The increasing number of clinical studies over time suggested also the extraordinary effectiveness of this blend for tissue regeneration/restoration, which has driven their investigation for the treatment of an even more broader range of diseases.

Thus, this review exclusively discussed the clinical efficacy of PRP or PRF collagen blends for regenerative medicine applications. In particular, PRP/Coll and PRF/Coll types were analyzed according to their manufacturing methods. Relative applications were discussed as well. All data were considered in order to underline the real advantages that lie in the combinatory use of PRP or PRF and collagen. Adverse events, regulatory aspects, and concerns were also reasoned.

## 2. Methods

This study was conducted according to PRISMA guidelines (https://www.prisma-statement.org). The review protocol was prospectively registered in the PROSPERO database (https://www.crd.york.ac.uk/PROSPERO), with the registration number 1089286. The research framework was defined by applying the PICO strategy. In particular, it was defined as follows: patients with acute or chronic conditions (P, Population); application of collagen combined with platelet concentrates (I, Intervention); conventional treatment, collagen alone, platelet concentrates alone or alternative regenerative approaches (C, Comparison); clinical outcomes including as pain reduction, functional recovery, healing rate (O, Outcomes). The generalization of P was intentional, as this systematic review aimed to include all studies investigating the use of PRP/PRF in combination with collagen, regardless of the specific acute or chronic condition addressed.

### 2.1. Eligibility Criteria

Included studies were only clinical studies (RCTs, prospective/retrospective cohort studies, case series, case studies) evaluating the effect of platelet concentrates (PRP or PRF) and collagen—combination treatments—in humans for regenerative medicine applications. All articles that dealt with the comparison of the efficacy of PRP/PRF versus collagen alone were discarded. In vitro, animal‐only studies, reviews, meta‐analysis, or editorials were excluded. Studies without clear outcome measures or incomplete results were also excluded, as well as all studies for which the full text was not available. No time frame limitations were applied. Only studies published in English were included.

### 2.2. Information Sources and Search Strategy

PubMed (https://pubmed.ncbi.nlm.nih.gov, accessed in September 2025), Google Scholar (https://scholar.google.com, accessed in September 2025), and U.S. National Library of Medicine (https://clinicaltrials.gov/, accessed in September 2025) were used as electronic search engines for all studies related to PRP/Coll and PRF/Coll formulations. Keywords included were “PRP”, “platelet‐rich plasma”, “PRF”, “platelet‐rich fibrin”, “collagen”, “gelatin”, “injectable”, “scaffold”, “hydrogel”, “microparticles”, and “nanofibers”. Boolean operators (AND, OR) were used.

### 2.3. Selection and Data Collection Processes

All titles and abstracts were screened by two reviewers. Full texts of potentially eligible studies were retrieved and assessed independently. Then, data such as study design, blend type, patients number, intervention details, outcomes, follow‐up, adverse events, and PRP/PRF preparation methods were extracted. The selection and data collection processes were manual. No automation tools were used.

### 2.4. Risk of Bias Assessment

The methodological quality of the included studies was systematically appraised by two reviewers, and disagreements were resolved through consensus. Results were summarized and graphically presented using the Robvis online tool [51] with a standardized summary graph.

### 2.5. Effect Measures

A qualitative narrative synthesis was performed due to heterogeneity in interventions and outcome measures. No meta‐analysis was conducted.

### 2.6. Synthesis Methods

Studies were grouped based on clinical indication and blend type (e.g., matrices, injectables, nanofiber, microparticles). Then, studies were grouped by clinical application: dental, musculoskeletal, peripheral nerve, bone regeneration, and ulcers. No statistical synthesis or meta‐analysis was performed due to high variability across studies.

### 2.7. Reporting Bias Assessment

Due to limited data and their heterogeneity, reporting bias was not formally assessed. Potential for publication bias was acknowledged based on study design and outcome reporting.

### 2.8. Certainty Assessment

A formal assessment of evidence certainty was not applicable. However, limitations of harvested evidence are described in the Discussion section based on study quality, size, and reporting.

## 3. Results

### 3.1. Study Selection and Characteristics

**Figure 2:**
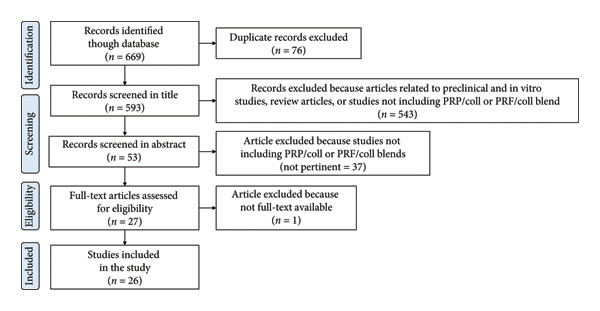
PRISMA guidelines‐based flowchart of the literature search and study selection process.

This review provides a cutting‐edge overview of all clinical studies performed on blood derivatives and collagen blends in order to explore their application fields and identify the most promising ones. PRISMA guidelines were followed. A total of 669 records were identified through database searching, of which 76 were removed as duplicates. Of the remaining 593, 543 were excluded for not meeting the inclusion criteria (e.g., articles related to preclinical and in vitro studies, review articles, or studies not including PRP/Coll and PRF/Coll blends). Then, 37 articles were excluded from the abstract screening step because of not pertinent to the aim of this study (studies not including PRP/Coll and PRF/Coll blends). Full texts of the remaining 27 articles were analyzed. One article was excluded because its full text was not available. Twenty‐six studies met the eligibility criteria and were included in the final qualitative synthesis. In the following sections, cited references correspond both to background studies and to studies that met the eligibility criteria described above. In order to increase manuscript clarity, studies selected according to eligibility criteria are listed in Table S1 and reported in Tables 1, 2, 3, and 4. The study selection process is detailed in the PRISMA 2020 flow diagram (Figure 2). The included studies were published between 2008 and 2025. Generally, four main categories of PRP/Coll and PRF/Coll were distinguished, based on the collagen formulation type. Indeed, collagen blends with blood derivatives were classified as matrices, injectables, micro‐ and nanoparticles, and nanofibers. These blends were involved in a variety of clinical applications, including dental (i.e., gingival recession and periodontitis), musculoskeletal (i.e., ligament and tendon injuries, bone regeneration), as well as for peripheral nerve regeneration, and for the treatment of chronic wounds. Only one investigation was registered for the treatment of fistula and cysts.

**Table 1:** Summary of the main characteristics of the included studies reporting the use of collagen combined with platelet concentrates (PRP/PRF).

Coll	PRP/PRF	Blood volume (mL)	Anticoag. type	Commercial kit	First CF (RPM; min)	Second CF (RPM; min)	Activation method	PRP/PRF vol. (mL)	Ref.
Matrix	PRP	20	N.d.	SmartPReP‐2 System, Harvest Technologies, Plymouth, MA.	2500; 1–3	2300; 6–9	N.d.	N.d.	[52]
		8.5	acid citrate dextrose	No	2400; 10	3600; 15	sodium alginate	0.3	[53]
		N.d.	N.d.	N.d.	N.d.	N.d.	N.d.	2	[38]
		20	N.d.	SmartPReP‐2 System, Harvest Technologies, Plymouth, MA.	2500; 1–3	2300; 6–9	autologous thrombin	3	[54]
		9	sodium citrate	No	1500; 15	No	calcium chloride and autologous thrombin	N.d.	[39]
		60	N.d.	Accelerate® Autologous Platelet Concentrating System, Exactech, Gainesville, USA https://www.exac.com	3800; 1.5	3800; 5	N.d.	10‐gen	[55]
							
		30	sodium citrate	TriCell PRP KIT, REV‐MED Inc., South Korea, https://www.tradekorea.com	3000; 4	3300; 3	thrombin	1.5–2.0	[56]
		400	ACD‐A	No	2000 g; 2	4000 g; 8	thrombin and calcium gluconate	51	[57]
		55	sodium citrate	Zimmer Biomet Gravitation Platelet Separation III (GPS III) kit (Zimmer Biomet, Warsaw, Poland) https://www.zimmerbiomet.com	3200; 15	No	bovine thrombine	6	[58]
	PRF	20–40	No	No	702; n.d.	No	No	1–2	[59]
		40	No	No	4500; 14	No	No	N.d.	[60]
		No	No	N.d.	N.d.	No	No	N.d.	[61]
		20	No	No	700; 3	No	No	2	[44]
		10	No	No	700; 3	No	No	N.d.	[48]
		20	No	No	2700; 12	No	No	N.d.	[62]
		20	No	A‐PRF 12, Process, Nice, France https://www.a-prf.com	600; 8	No	No	N.d.	[45]
		N.d.	No	IntraSpin, Intra‐Lock System Europa, Salerno, Italy, https://www.intra-lock.com	2700; 3	No	No	N.d.	[49]
		10	No	No	3000; 12	No	No	N.d.	[63]
		10	No	No	2700; 12	No	No	N.d.	[64]
		N.d.	No	No	3000; 10	No	No	N.d.	[65]

Injectable	PRP	N.d.	N.d.	Tropocells Plus, Estar Medical, Holon, Israel https://www.tropocells.com	N.d.	No	No	2.7	[16]
			N.d.	No	1500; n.d.	No	N.d.	1.7	[42]
			ACD‐A, sodium citrate	GLO PRP, GLOFFIN America LLC, NJ, USA https://www.glofinn.com	1800; n.d.	1900 g	No	2	[15]
		15	N.d.	No	1500; 5	No	N.d.	N.d.	[47]

Particles	PRF	8	No	No	700; 3	No	No	N.d.	[46]

Nanofibers	PRP	N.d.	N.d.	N.d.	N.d.	No	N.d.	N.d.	[43]

*Note:* The type of collagen employed (Coll), the platelet concentrate used (PRP/PRF), the initial blood volume (mL) processed, the anticoagulant type (if any), the commercial kit adopted for preparation, and the centrifugation protocols including the first centrifugation force (CF; RPM and minutes) and second centrifugation force (RPM and minutes), when applicable. In addition, the activation method used for platelet concentrate preparation, the final PRP/PRF volume obtained (mL), and the corresponding reference (Ref.) for each study are reported. All blood derivatives were obtained by means of centrifugation.

**Table 2:** Summary of included studies by pathology, platelet concentrate type, leukocyte content, and the corresponding reference (Ref.).

Pathology		Platelet concentrate type	Leukocyte content	Ref.
Dental and periodontal disorders	Gingival recession [52]	PRP	LR	[52]
	Peri‐implant gingiva insufficiency [61]	PRF	LR	[61]
	Chronic apical periodontitis [53]	PRP	LR	[53]
	Maxillary sinus lift [54]	PRP	LR	[54]
	Maxillary sinus membrane perforation [62]	PRF	LR	[62]
	Radicular cyst [65]	PRF	LR	[65]
	Chronic oroantral fistula [60]	PRF	LR	[60]
	Root‐end surgery [59]	PRF	LR	[59]
	Periodontal intrabone defects [63]	PRF	LR	[63]
	Periodontal intrabone defects [64]	L‐PRF	LR	[64]
	Gingival recession defects [48]	PRF	LR	[48]
	Single gingival recession [45]	PRF	LR	[45]
	Gingival recession [44]	PRF	LR	[44]
	Multiple gingival recessions [49]	PRF	LR	[49]
	Periapical bone defects [46]	PRF	LR	[46]

PNI	Traumatic peripheral nerve injuries [56]	PRP	LR	[56]
	Chronic peripheral nerve pain [58]	PRP	LR	[58]

Musculoskeletal disorders	Temporomandibular joint ankylosis [38]	PRP	N.d.	[38]
	Anterior cruciate ligament reconstruction [55]	PRP	LR	[55]
	Chronic lateral epicondylitis [16]	PRP	LP	[16]
	Lateral epicondylitis [42]	ACP	LP	[42]
	Shoulder impingement syndrome [47]	ACP	N.d.	[47]
	Partial‐thickness rotator cuff injuries [15]	PRP	LR	[15]

Chronic wounds	Diabetic foot ulcers [43]	PRP	N.d.	[43]
	Pressure sores [39]	PRP	LP	[39]
	Recalcitrant chronic ulcers [57]	PRP	LP	[57]

Abbreviations: LP, leukocyte‐poor; LR, leukocyte‐reach; PNI, peripheral nerve injuries.

**Table 3:** Overview of the clinical studies included in the review on PRP/Coll matrices.

Type of study	Pathology	Treatment (*n*)	Control (*n*)	Blood derivative	Follow‐up (months)	Control	Treatment	Main outcome(s)	Ref.
RCT	Gingival recession	9	9	PRP	4	ADM	PRP + ADM	Root coverage of 90% (control: 70%).	[52]

Case series	Gingival recession	6	—	PRF	6	—	PRF + Coll	RH 2.4 ⟶ 0.7 mm; RW 4.1 ⟶ 1.8 mm; RC%: 71.7%; CAL 3.5 ⟶ 1.8 mm; gingival biotype improved; VAS‐E ≈9/10	[48]

RCT	Gingival recession	13	13	PRF	6	Coll	PRF + Coll	Both groups showed significant reduction in RD and RW; greater increase in keratinized tissue width and higher percentage of root coverage in the PRF + Coll group.	[44]

Case report	Single recession	1	—	PRF	6	—	PRF + Coll	Complete root coverage (4 mm); gingival 1 mm thicker; uneventful healing	[45]

Case report	Multiple recessions	1	—	PRF	30	—	PRF + Coll	83% mean root coverage at 12 months; no donor morbidity	[49]

Case report	Peri‐implant deficiency	1	—	PRF	1	—	PRF + Coll	Gingival width from 0.5 to 3–4 mm; thickness 4.5 mm.	[61]

RCT	Apicomarginal defects	9	10 (Coll) 6 (PRP)	PRP	12	Coll or PRP	PRP + Coll	Healing rates: 80% (Coll), 83% (PRP), 89% (PRP + Coll). Reduction in probing depth in all groups, improvement in attachment level, gingival margin elevation, reduction in lesion size and periapical rarefactions.	[53]

RCT	Maxillary sinus lift	10	10	PRP	8	PRP + xenograft	PRP + Coll	Satisfactory increase of bone height in all groups. Greater maturation was observed in PRP group with the formation of orderly lamellar bone, while in the control group, woven bone prevailed with a disorganized arrangement of collagen fibers.	[54]

Case report	Sinus membrane perforation	1	—	PRF	6	—	PRF + Coll	Successful sealing; stable graft; sufficient bone for implant.	[62]

RCT	Root‐end surgery	25	25	L‐PRF	0.25	Coll	PRF + Coll	No differences in pain/swelling and improvement; no complications	[59]

RCT	Intrabony defects	14	14	PRF	6	HA + Coll + PLA–PGA	PRF + Coll + HA	Both groups showed significant reduction in probing depth, clinical attachment gain and radiographic bone filling; no significant differences emerged between the treatments.	[63]

RCT	Periodontal defects	20	10	L‐PRF	6	Debridement and PRF	PRF + Coll	Radiographic defect reduction greater with PRF + Coll (33%); similar clinical gains	[64]

RCT	Pressure sores	160	160	PRP	1.75	PRP + gelatin	PRP + Coll	Both groups achieved similar healing rates (83%–86%). Lesion depth was the only predictor of healing speed. A significant improvement in quality of life was observed in both groups, with no adverse events.	[39]

Case series	Chronic ulcers	10	—	PRP	3	—	PRP + fibrin glue + Coll	Complete healing; mean closure 11 weeks.	[57]

Case series	Ankylosis	19	—	PRP	18	—	PRP + Coll + HA	Improved opening; no recurrence; radiographic neocondyle.	[38]

Case series	Ligament reconstruction	14	—	PRP	24	—	PRP + Coll	No failures at 2 years; intact grafts; safe.	[55]

Comparative study	Peripheral nerve trauma	16	11	PRP	185	Autograft	PRP + Coll + autograft	Autograft: pain 8.6 ⟶ 0.27; 81.8% elimination. PRP repair: pain 7.7 ⟶ 0 within 2 months	[58]

RCT	Peripheral nerve injuries	110	112	PRP	6	Epineurotomy	Coll + PRP + Epineurotomy	Electrophysiological recovery; sensory/motor benefit in median nerve; Overall functional evaluation did not significantly differ between groups; No severe complications.	[56]

Case report	Radicular cyst	1	—	PRF	5	—	PRF + Coll + xenograft	Bone regeneration at 12–20 weeks; tooth stability restored; esthetic crown.	[65]

Case report	Oroantral fistula	1	—	PRF	1.5	—	PRF + Coll	Complete closure at 2 weeks; tissue thickening; no recurrence.	[60]

*Note:* The table summarizes the type of study design, the pathology addressed, the number of patients enrolled in the control group and in the treatment group (*n*), and the type of blood derivative (PRP, PRF) employed. The follow‐up period (months) is reported alongside the characteristics of the control and treatment groups. The main clinical outcomes are highlighted to provide a comparative view of therapeutic effectiveness. RC%, percentage of root coverage; RC%, percentage of root coverage; VAS‐E, visual analog scale for aesthetics; HA, hydroxyapatite.

Abbreviations: ADM, acellular dermal matrix; CAL, clinical attachment level; PLA–PGA, poly(lactic acid)‐poly(glycolic acid); RD, recession depth; RH, recession height; RW, recession width.

**Table 4:** Overview of the clinical studies included in the review on PRP/Coll injectables.

Type of study	Pathology	Treatment (*n*)	Control (*n*)	Blood derivative	Follow‐up (months)	Control	Treatment	Main outcomes	Ref.
Prospective trial	Lateral epicondylitis	40	—	PRP	6	—	PRP + Coll	Safe; PRTEE ↓59%; SF‐12 ↑30.7 ⟶ 37.7; grip ↑28.8 ⟶ 36.8 kg; sonographic healing in 68%	[16]

RCT	Partial‐thickness rotator cuff injury	30	30 (Coll) 30 (PRP)	PRP	6	Coll/PRP	PRP + Coll	No significant differences; trend of improvement in PRP groups (12–24 weeks); cuff regeneration in all groups	[15]

Prospective pseudo‐RCT	Shoulder impingement	29	29	ACP	6	Corticosteroid	ACP + Coll	CMS 83.8 vs 78.5; DASH 12.4 vs 18.9; SST 10.8 vs 9.7; strength 6.8 vs 5.9 kg. ACP + Coll gave superior function and strength	[47]

Case series	Lateral epicondylitis	5	—	ACP	2.5	—	ACP + rhColl	VAS pain 6.4 ⟶ 1.8; ROM preserved; MRI showed tendon healing in 4/5	[42]

*Note:* The table summarizes the type of study design, the pathology addressed, the number of patients enrolled in the control group and in the treatment group (*n*), and the type of blood derivative (PRP, PRF) employed. The follow‐up period (months) is reported alongside the characteristics of the control and treatment groups. The main clinical outcomes are highlighted to provide a comparative view of therapeutic effectiveness. SF‐12, 12‐Item Short Form Health Survey; CMS, Constant–Murley Score (shoulder function).

Abbreviations: ACP, Autologous Conditioned Plasma; DASH, Disabilities of the Arm, Shoulder, and Hand; MRI, Magnetic Resonance Imaging; PRTEE, Patient‐Rated Tennis Elbow Evaluations; ROM, Range of Motion; SST, Simple Shoulder Test; VAS, Visual Analog Scale.

**Figure 3:**
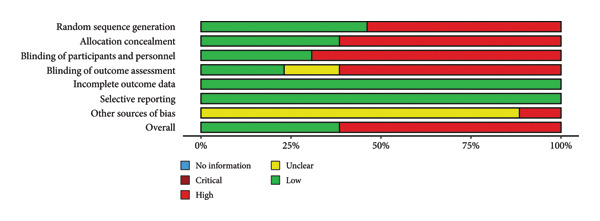
Risk of bias assessment across all the included studies.

PRP/PRF formulations varied in preparation protocols and concentrations as well as collagen, which was applied as a porous membrane, a nanofibrous matrix, or as injectable hydrogel. A risk of bias tool was applied in order to highlight study limitations (Figure 3). Most studies had methodological limitations such as small sample sizes, lack of control groups, nonrandomized designs, or short follow‐ups. All these factors were considered in the qualitative and critical interpretation of the results.

### 3.2. PRP/Coll Blend Types

Blood derivatives and collagen blends were developed in order to improve regenerative medicine approaches clinical outcomes, following the belief that the combination of the properties of two biomaterials could enable the development of a device with excellent bioactivity. Moreover, the application of this strategy could help overcome some drawbacks that lie in the use of blood‐derived products. PRP lacks a distinct morphology and displayed a nonuniform distribution of its constituents [18]. Thus, PRP incorporation in a matrix can bring additional advantages such as a more homogeneous distribution and controlled release over time, which could result in higher effectiveness. According to the literature, impregnation or incorporation of PRP into a 3D collagen matrix helps overcome this issue, as shown by histological examinations of PRP/Coll scaffolds [18].

PRP acts locally and immediately at the target site, and its bioactive role is confined to a few days. The incorporation of PRP in a scaffold allows for its controlled release over time, a strategy that enhances its regenerative properties by providing pro‐regenerative growth factors release for weeks [18, 19, 27, 48]. Indeed, growth factors could be released from PRP‐impregnated collagen scaffolds up to 14 days and in vitro promote cells proliferation, angiogenesis, vascularization, and anti‐inflammatory processes to a greater extent than collagen scaffolds alone [18].

An indirect advantage of incorporating PRP in collagen scaffolds also relies in the structural stability that the construct gains. PRP has very low resistance to physiological stresses, an intrinsic property that limits its applications. Thus, the impregnation of collagen scaffolds with PRP provides PRP with the desired architecture and morphology.

PRP/Coll blends were developed for several applications and manufactured according to specific clinical needs. In particular, PRP was found to be incorporated or impregnated in 2D or 3D collagen‐based scaffolds in the form of hydrogels, sponges, injectable suspensions, nanofibrous matrices, or microparticles. Different manufacturing techniques were employed according to the application (Table 2). Indeed, porous matrices were mainly used for the treatment of gingival recessions, periodontitis, and ulcers. In this case, both PRP/Coll and PRF/Coll blends were tested. Contrarily, Coll injectable formulations were used for tendon and ligament defects, in mix only with PRP. More experimental were nanofibrous matrices and microparticles, which were developed only for the treatment of ulcers and for bone regeneration, respectively. Only one clinical study investigating PRP/collagen nanofibers was conducted in 2021, and another focusing on PRF/collagen microparticles was reported in 2023. In the following sections, PRP/Coll and PRF/Coll blend types were discussed in detail. Their development techniques, as well as their properties and clinical efficacy, were detailed.

In assessing the efficacy of PRP/Coll and PRF/Coll blends, one of the most critical aspects concerns the intrinsic variability of platelet‐rich preparations, which is strongly influenced by both the type of concentrate (PRP vs. PRF and their subtypes) and the specific obtaining procedures employed. As summarized in Table 1, a variety of centrifugation‐based protocols have been applied across studies, resulting in products that are superficially classified under the same terminology, but they differ substantially in their cellular composition, platelet concentration, and growth factor content. These discrepancies profoundly affect the biological activity of the autologous material, thereby introducing significant heterogeneity in treatment outcomes.

Additionally, the standardization of outcomes becomes a challenge also because no clear information is provided not only about platelet concentration but also about volume used (both the volume collected and the volume used for the preparation of blends). Similarly, the PRP/Coll and PRF/Coll ratio is never reported. The lack of harmonization in preparation methods not only limits the reproducibility of results but also hinders the possibility of drawing reliable comparisons across clinical studies, making it difficult to delineate the real therapeutic potential of PRP/Coll systems. Furthermore, the absence of universally accepted classification and reporting standards exacerbates this issue, leading to a situation where the same product label may conceal different biological formulations. This methodological variability directly translates into challenges in standardizing outcome measures, ultimately impeding the establishment of evidence‐based guidelines. Therefore, the development and adoption of standardized protocols for PRP/PRF preparation and characterization represent a crucial step toward achieving reliable and translatable clinical evidence on PRP/Coll and PRF/Coll blends.

Another critical aspect concerns the source of collagen extraction and the methods employed in product manufacturing, which can profoundly influence clinical outcomes. Collagen may be derived from bovine, porcine, equine, marine, or recombinant sources, each characterized by distinct biochemical profiles and immunogenic potential. Moreover, variations in purification, crosslinking, sterilization, and formulation processes affect collagen’s structural integrity, degradation kinetics, and biological performance. Such heterogeneity can result in differences in tissue integration, host response, and long‐term efficacy, thereby limiting the comparability of clinical data across studies. As with PRP/PRF, the lack of standardized protocols and detailed reporting hampers reproducibility and prevents robust conclusions regarding the true therapeutic value of collagen‐based interventions.

Leukocytes play a central role in modulating inflammation, antimicrobial defense, and early tissue remodeling. Their high or low concentration in PRP/Coll and PRF/Coll preparations is thus a clinically relevant aspect. Given the substantial heterogeneity in PRP/PRF preparation methods observed across the included studies, understanding the expected leukocyte profile of each formulation is essential for a deep interpretation of clinical outcomes. For clarity and to facilitate comparison between studies, each study included was classified according to pathology, type of platelet concentrates (PRP or PRF) and its expected leukocyte content (LP or LR) in Table 2. Although leukocyte data were rarely reported explicitly, the preparation protocols summarized in Table 1 allow reliable inference of leukocyte content. Indeed, PRF protocols and PRP systems based on buffy coat separation or dual‐spin centrifugation are known to generate LR concentrates, whereas plasma‐based devices and low‐g single‐spin methods typically produce LP formulations [66–68]. This relationship is reflected in the distribution of formulations across applications: dental and periodontal treatments almost exclusively employed LR concentrates (mainly PRF), consistent with the need for controlled inflammatory activation and enhanced soft‐tissue healing; peripheral nerve applications likewise relied on LR‐PRP. Musculoskeletal studies used a mix of LR and LP preparations, reflecting differing therapeutic goals across tendon, ligament, and joint disorders. In chronic wound management, LP‐PRP was used more frequently, in line with the objective of limiting excessive or prolonged inflammation in ulcerated tissues.

#### 3.2.1. Matrices

Collagen 3D scaffolds, also referred to as matrices are commonly known as sponges, pads, or hydrogels and are usually developed through freeze‐drying or sol–gel transitions. The versatility of these manufacturing techniques and of collagen as material has allowed the production of matrices of the preferred size (i.e., with regular or irregular shape) and properties (e.g., stiffness, wettability, half‐life, porosity). The collagen types used for clinical studies included medical‐grade collagens derived from swine, bovine, or fish [39, 44, 45, 48, 49]. The collagen type and its properties can not only influence the blend bioactivity but also the PRP/PRF absorption in the matrix, an aspect that must be considered prior to selecting the ideal scaffolding source. Moreover, not only collagen extraction source but also its processing and manufacturing can affect the wettability of the final product. As reported in a recent study about the impregnation power of porcine‐derived collagen matrices, highly porous 3D sponges were found to be fully hydrophilic while less porous or more dense matrices were partially or only superficially wettable [69]. Products developed from materials with highly preserved natural crosslinks, as in the case of insoluble collagen fibers, were instead found to not be able to absorb PRP/PRF‐like materials [69].

Two enriching strategies were adopted: pre‐ and post‐processing (Figure 4). Usually, the post‐processing method is adopted, where blood derivatives were impregnated into ready‐to‐use freeze‐dried sterile collagen porous sponges [17–19, 45, 48, 49]. Only a few works mixed PRP with collagen suspensions prior to gelification [20, 28, 30]. In all cases, blood derivatives were isolated from the patient’s own blood and mixed with collagen immediately before use. However, the volume of loaded PRP or the PRP/Coll weight ratio was not clearly specified. Scaffolds were generally impregnated with PRP until fully saturated. In order to standardize the role of PRP in PRP/Coll blends and allow for reproducible results across multiple medical teams or research groups, more accurate data must be reported.

Figure 4Pre‐ (a) or post‐(b) processing addition of PRP to collagen. Picture (a) was reproduced with permission (2016, Wiley Online Library) [20]. Picture (b) was reproduced under terms of the CC‐BY license (2023, published by Hindawi Publishing Corporation) [49].

**Figure:**
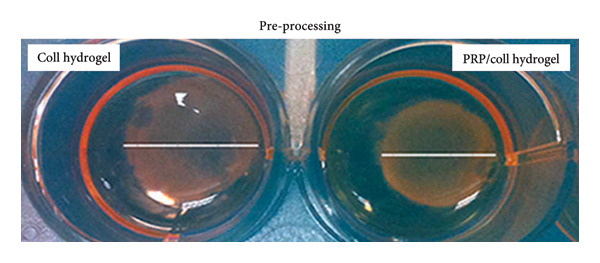
(a)

**Figure:**
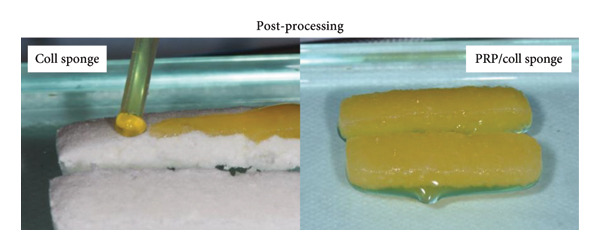
(b)

Clinically, PRP/Coll and PRF/Coll matrices have been tested only for gingival recession [44, 45, 48, 49, 52], the treatment of severe wounds [39], ligaments repair [55], peripheral nerve injuries [56, 58], bone regeneration [38, 41], cyst [65], ankylosis [38], and fistula [60] (Table 3).

The restoration of the protective role of the gingiva but also of the esthetic balance between soft tissues and adjacent tooth structures is the request of patients and the main objective of clinicians. Currently, the gold standard strategy for severe gingival recession healing is autografts. However, well‐known drawbacks of autografts have driven the search for less painful, time‐consuming, cost‐effective, and equally resolutive strategies. Although positive, biomaterials‐based approaches (e.g., collagen scaffolds) are still not as resolutive as autologous tissues grafts. To enhance the efficacy of xenografts, PRP/PRF addition could be a promising solution. Collagen matrices and blood derivatives separately proved to be quite effective for recession defects [70, 71], with comparable outcomes (NCT05988086) [50]. To date, there is little evidence of their advantageous combined effect [45, 48, 49, 52] since their efficacy on gingival recessions has only recently begun to be investigated, starting in 2022. Although with many limitations (e.g., short follow‐up periods, absence of control groups, small number of enrolled patients), all performed studies had positive outcomes. One of the earliest randomized controlled clinical pilot studies involved 18 patients with Miller Class I or II gingival recession defects (≥ 3 mm) treated either with a coronally positioned tunnel (CPT) plus an acellular dermal matrix allograft (ADM) or with CPT/ADM/PRP (CPT/PRP) [52]. After 4 months, the PRP‐treated group achieved 90% defect coverage compared to 70% in the control group [52]. Subsequently, a comparative study was conducted by Patra et al., on the efficacy of PRF/Coll membranes compared to Coll membranes alone in 12 patients (CTRI/2020/06/026141) [44]. After 6 months of treatment, about 25% of the sites of the PRF/Coll group reached complete root coverage while only 5% of the sites showed complete root coverage in the control group (Coll membrane alone) [44]. Following this study, three case reports confirmed PRP/Coll blends efficacy. The case report presented by Santamaria et al. showed complete gingival restoration after 6 months [45]. The case report by Michels et al. demonstrated 83% root coverage of multiple gingival recessions at 12 months post‐treatment, with sustained preservation of the keratinized mucosa band following the application of a porcine‐derived sponge impregnated with PRF [49]. Lastly, similar outcomes were observed on six cases by Krishnaraj et al. six months post‐application of a PRF‐infused marine collagen scaffold [48]. Those findings suggest the potential of blood derivatives combined with collagen for root coverage in single and multiple gingival recession cases in overcoming limitations of autogenous connective tissue grafts (e.g., postoperative discomfort in the donor area, restrictions in the graft size, surgeon expertise, additional required personnel) [48, 49]. The use of xenogeneic substitutes (well established in clinical practice) [23] would thus overcome those limitations. Even though donor site access for connective tissue graft would not be necessary in the PRP/Coll approach, it is important to remember that, although limited, blood collection from patient increases the number of clinical steps during the surgery and requires the presence of specialized medical personnel (e.g., nurse to collect blood and handle it).

Regarding ankylosis, positive outcomes were achieved by combining the osteoconductive/osteogenic power of collagen, hydroxyapatite and PRP, which led to significant improvement in mouth opening in about 19 patients with no regression up to 18 months [38]. Although preliminary and limited (e.g., small number of participants, lack of control groups), these results suggest the potential of the combination of these materials for this application.

PRF/PRP used together with Coll membranes can significantly enhance soft tissue augmentation and wound closure, for instance, in peri‐implant keratinized gingiva augmentation [61], oroantral fistula repair [60], and sinus membrane perforation management [62]. Moreover, randomized controlled trials indicate that PRF/Coll promotes bone fill and clinical attachment gain in intrabony defects [63, 64]. In the context of apicomarginal defects, PRP, PRP combined with a Coll sponge, and a Coll membrane alone efficacy was tested in 30 patients with chronic apical periodontitis [53]. After 12 months, combined clinical–radiographic healing occurred in 80% of the Coll group, 83.3% of the PRP group, and 88.9% of the PRP/Coll group, with all treatments producing significant reductions in probing depth, clinical attachment level, gingival margin position, and periapical lesion size [53]. Similarly, in endodontic and periradicular surgery, collagen membranes associated with PRP or PRF have been reported to improve healing of apicomarginal defects and to support periapical tissue regeneration [59]. Furthermore, in sinus lift and augmentation procedures, PRP combined with collagen scaffolds has been associated with improved bone maturation and radiographic outcomes [54]. Finally, in cystic lesion management, the synergistic use of PRF, demineralized bone matrix, and type I collagen resorbable membranes has demonstrated effective bone regeneration and defect resolution [65].

Even more experimental was the clinical testing of PRP/Coll matrices for the healing of severe wounds [39]. To the best of our knowledge, two clinical studies have been published. In particular, one dealt with the comparative evaluation of PRP applied in debrided pressures sores and covered with a gelatin hydrogel or a collagen sponge. Although outcomes of this randomized clinical trial were not statistically significant, PRP/Coll combination healed pressure sores in less time compared to PRP/gelatin (PRP/Coll: 5 weeks, PRP/gelatin: 6 weeks) [39]. Results suggested the higher efficacy of native collagen but reported data did not allow us to confirm it because of the lack of information about the collagen nativeness degree. Moreover, the slightly lower healing time revealed in the case of PRP/gelatin could be due to residues of glutaraldehyde used for the hydrogel development, that although neutralized could have a negative impact on sores regeneration. Additionally, the study is too limited to assess the real effectiveness of PRP/Coll blends in severe wounds healing. The second study, being a Case series do not allow us to strength outcomes of the previously analyzed study. However, even if a control group is missing, it should be accounted that PRP/Coll combination allowed for a wound complete closure in 9/10 patients [57].

More accurate clinical trials should be performed, preferably with PRP‐impregnated collagen matrices, on an adequate number of participants. The lack of data suggested highlights the unexplored potential of this field of application and the need of in‐depth investigative analysis.

#### 3.2.2. Injectables

Collagen‐based injectables are produced by resuspending ad hoc prepared collagen forms in neutral buffered solutions [26]. Collagens used for medical‐grade injectable PRP/Coll blends development were derived from porcine skin [15] or recombinant systems [16, 42, 47]. The manufacturing processes of collagen injectable preparations are not accessible because they belong to companies know‐how. Commercial collagen injectable formulations are provided in dry form or in a hydrated, ready‐to‐use state at 4°C. In the first case, collagen formulations must be rehydrated in saline solution (NaCl 0.9%, w/v) before use. Freshly isolated PRP is mixed with collagen injectable preparations by means of commercial or in‐house double syringe systems for few minutes, before being immediately injected into the target area.

Harvested clinical studies on PRP/Coll injectables were found to be only four [15, 16, 42, 47]. In these studies, PRP was mixed both with dry Coll injectable preparations [16, 42, 47] and with already resuspended Coll preparations [15]. The use of PRP/Coll injectables is relatively recent. Preclinically, PRP/Coll injectables have been evaluated only for the treatment of arthritis [32]. The first clinical trial in which their efficacy has been evaluated dates back to 2019 [16]. From then, they were tested for the resolution of tendinopathies, in particular epicondylitis [16, 42] and rotator cuff tears [15, 47] (Table 4). The first study conducted on 40 people affected by lateral epicondylar tendinopathy registered about 25% reduction in Patient‐Rated Tennis Elbow Evaluation scores 6 months after a single treatment in almost all enrolled patients (about 86%) [16]. However, although encouraging results, this study lacked a control group which did not allow for a proper evaluation of the trial outcomes. Similar outcomes were reported in a later study, in which the treatment of five cases of chronic epicondylitis with a single injection of PRP/rhColl led to extensive long‐term healing (complete or regression) even in patients with chronic symptoms for about 10 months [42]. A similar investigation was done with PRP/rhColl for the treatment of shoulder impingement syndromes (including rotator cuff tendinopathy or partial‐thickness rotator cuff tears) [47]. This prospective pseudo‐randomized trial involving 29 patients revealed the superior recovery of tendon strength compared to the standard corticosteroid injection, suggesting that PRP/Coll could be at least as beneficial as conventional therapies but without typical side effects associated with steroids use (e.g., allergic reactions, tissue necrosis) [47]. Moreover, the execution of a single treatment (one injection instead of three, as commonly practiced) could significantly decrease the infection rate and patients’ stress, as well as improve their well‐being. Consequently, PRP/Coll single injections could decrease treatments costs by reducing outpatient clinic visits that involve disposables, instruments, as well as qualified personnel [47]. Moreover, the 3D supporting scaffold is expected to provide longer retention and bioavailability of growth factors, thereby preventing their early wash‐out from the injection site. The exploitation of PRP/Coll for the treatment of partial thickness rotator cuff injuries was evaluated also by Godek et al. in a single‐center open randomized controlled trial on 90 patients treated with PRP alone, swine collagen/iris alone, and PRP/Coll/iris injections (NCT: NCT04492748) [15]. Although good outcomes were reached by all three experimental groups (single components and blend), their findings revealed not statistically significant differences among treatments efficacies. According to their study, the combined therapy of PRP and collagen was found, in that case, not to be more effective than monotherapies in reducing pain symptoms or improving mobility, self‐care, or usual activities [15]. In this case as well, the real effectiveness of PRP/Coll injectables requires a more in‐depth investigation with a higher number of enrolled patients. Although promising, four clinical studies are not enough to confirm the superior efficacy of PRP/Coll blends against conventional therapies for the treatment of tendinopathies.

More experimentally, PRP was also mixed with collagen in the form of dry powder/particles in order to obtain nonflowable paste to be applied in irregular bone defects. Although limited to a single clinical study, the work of Arpitha et al. reported nearly complete bone healing with a PRF/Coll paste (90% collagen, 2% Mupirocin, 1% Metronidazole) compared to control, underlining the absence of advantages in using an advanced medication instead of applying traditional methodologies for this particular application (NCT04391725) [46].

#### 3.2.3. Micro‐ and Nanoparticles

Approximately 70% of PRP growth factors are released within 10 min and nearly 100% within 1 h after injection [1]. Although some attempts to delay and control factors release were made using PRF matrices, they revealed to be insufficient. Due to this, advancements were made toward the development of controlled‐release systems of PRP by means of collagen‐based micro‐ or nanoparticles. Although research on PRP/PRF‐loaded microparticles is ongoing, no clinical studies have yet been performed on PRP/PRF‐loaded collagen‐based controlled‐release systems. A single randomized controlled clinical trial investigated the potential of PRF and empty collagen particles (unknown source and manufacturing method) in the management of through‐and‐through periapical defects following endodontic microsurgery compared to periapical surgery [46]. In this case, PRF was loaded in collagen particles by absorption rather than encapsulation. Outcomes registered after 12 months on 38 patients reported a complete closure of the cortical window in both groups, with no statistically significant differences observed between the experimental and control cohorts across clinical, radiographic, or volumetric measure. Although limited to a single trial, these findings suggested the absence of a measurable clinical advantage in the use of PRF combined with collagen compared to microsurgery alone [46].

#### 3.2.4. Nanofibers

Advancements were made also toward the controlled release of PRP from collagen‐based nanofibers. To date, only one randomized clinical trial has investigated the use of PRP‐loaded collagen nanofibrous matrices for the treatment of diabetic foot ulcers on 28 patients, highlighting the very limited clinical evidence available in this field [43]. A porcine skin–derived gelatin‐based nanofibrous matrix was developed and applied to the wound area with or without human placenta‐derived mesenchymal stem cells after PRP gel application in the diabetic foot ulcer bed (IRCT20190214042712N1) [43]. Although limited, a significant difference was registered in the PRP‐enriched group in accelerating wound closure (71% of wound size reduction instead of 66% of the control group) [43]. These findings indicate that, despite the promising rationale for combining PRP with nanofibrous matrices, clinical outcomes remain inconclusive and require further validation through more and well‐designed clinical trials.

Preclinical evidence may help to contextualize these results. A limited number of in vitro studies have attempted to load PRP into electrospun nanofibers (three studies). PRP was mixed with polycaprolactone/gelatin (unknown extraction source), polyvinylidene fluoride/collagen (unknown extraction source), or nanohydroxyapatite/poly(lactic acid)/gelatin before being subjected to 12–21 kV, the voltage necessary to produce 300–1000 μm diameter nanofibers [72–74]. In vitro results on human bone marrow mesenchymal stem cell revealed non–statistically significant positive outcomes, suggesting how that processing parameters (e.g., applied voltage, solvents, working temperature, sterilization) could affect PRP integrity and thus bioactivity [72]. Conversely, another study reported continuous PRP release during the observation time (14 days) as well as positive outcomes not only in terms of biocompatibility but also enhanced osteogenic differentiation [73]. However, concerns remain regarding the solvents used, whose toxicity limits their clinical translation, since traces of them could negatively affect the biological response of the devices. The known issues of techniques used for nanofibers development (i.e., solvents type and concentration, working temperature, process time, applied voltage) suggested that it should be better to add PRP with collagen scaffolds at the end of their production process, including sterilization [43, 74].

## 4. PRP/Coll‐Related Adverse Events

Collagen is one of the most preserved structural proteins of vertebrates, whose amino acid sequence is quite unchanged across animal species and centuries, suggesting its fundamental role for cells viability and interaction. According to this, low immune reactions have even been registered against xenogeneic collagen, depending on the protein extraction source [23]. PRP/PRF is a blood derivative isolated from patients’ own blood and immediately injected/applied for the treatment of injured body districts of the same patients. Due to this, immune reactions or adverse events have never been reported, except when related to other causes (e.g., wrong surgical procedures, infections).

Of the 26 included clinical studies, only five (28%) explicitly reported adverse events. The absence of such data likely could reflect underreporting as well as presence [16, 38, 46, 47, 59] or absence [39, 43, 47, 54–58, 70] of complications, limiting the ability to assess the overall safety of PRP/Coll and PRF/Coll blends, underscoring the need for standardized reporting in future studies. Among the studies that reported safety outcomes, no serious adverse events were observed, no patients discontinued treatment due to complications, and no implant failures were recorded [16, 46–48]. Reported events were mild and self‐limiting, primarily associated with the surgical intervention. For instance, in patients treated with pads, no edema or swelling was noted in the first 14 days postoperatively [45]. Swelling was registered by Meschi et al. in 1/25 patients [59]. In cases involving injectables, approximately 70% of patients reported transient post‐injection pain, which resolved within 4 h [47]. Pain or swelling at the injection site that occurred after the injection in 20/40 in another study [16]. Similarly, the application of a paste for bone regeneration was associated with mild pain and swelling, but with no statistically significant differences between experimental and control groups [46]. In a pilot study on temporomandibular joint ankylosis, mild fever and transient facial nerve involvement were observed in a few patients, all of which resolved spontaneously without long‐term consequences [38]. In the evaluation of adverse events, it should also be considered that complications may not necessarily arise from the biomaterial itself, but rather from the surgical procedures itself. Factors such as surgical technique, intraoperative handling, postoperative care, and patient‐specific variability (e.g., comorbidities, compliance) can significantly contribute to the occurrence of adverse events. Distinguishing between device‐ or product‐related complications and those attributable to procedural aspects is therefore essential to accurately interpret safety profiles and to avoid overestimating the risks associated with the investigated biomaterial.

## 5. Risk of Bias Assessment

Figure 3 summarizes the risk‐of‐bias evaluation across the included studies and highlights several recurrent methodological limitations that were carefully considered in the interpretation of the evidence. A substantial proportion of studies showed high or critical risk in the domains related to random sequence generation and allocation concealment, mainly due to the prevalence of nonrandomized designs and the frequent lack of detailed methodological reporting. Similarly, blinding of participants, personnel, and outcome assessors was rarely feasible for procedural reasons or not explicitly documented, resulting in predominantly high or unclear risk in these categories. In contrast, most studies were judged at low risk for complete reporting, as follow‐up and outcome measures were generally well described. The domain addressing other potential sources of bias revealed additional limitations—including small sample sizes, absence of protocol registration, and heterogeneous baseline characteristics—reflecting the early stage of the clinical investigations in this field.

Overall, while the existing studies consistently reported favorable clinical outcomes, the methodological weaknesses identified in the risk‐of‐bias assessment underscore the need for more randomized controlled trials.

## 6. Regulatory Aspects and Concerns

Platelet concentrates, in combination with collagen, gained increasing attention in regenerative medicine due to their well‐documented clinical efficacy. However, their use is subjected to specific regulatory requirements, particularly regarding non‐transfusional blood derivatives, which establish the legal and procedural framework for blood derivatives collection, processing, and clinical applications. According to these regulations, PRP/PRF (autologous or allogeneic) can be used only under specific conditions. Autologous PRP/PRF‐based treatments can be performed either in transfusion centers or in authorized Facilities by Transfusion Services, providing greater accessibility and flexibility for patients. Conversely, allogeneic PRP/PRF‐based treatments are subject to stricter regulations since the entire blood management process, from blood collection to its final application, must be conducted exclusively within transfusion centers. While these requirements ensure rigorous quality control and patient safety, they also limit the use of allogeneic PRP/PRF, which is particularly advantageous in medical settings where transfusion centers are not readily available or when autologous blood is not a viable option (e.g., hematological disorders). Due to this, although it has significant clinical advantages, allogenic materials are rarely used. Furthermore, transfusion centers permit nontransfusional uses of PRP/PRF, regardless of its origin, solely when substantiated by strong scientific evidence. In the absence of sufficient clinical data, specific clinical trials must be conducted to demonstrate the safety and efficacy of the treatment before its routine application. In this framework, further supportive scientific supportive research and subsequent regulatory updates could facilitate a broader application of this innovative therapy, offering significant benefits for both patients and regenerative medicine advancements.

## 7. Discussion

**Figure 5:**
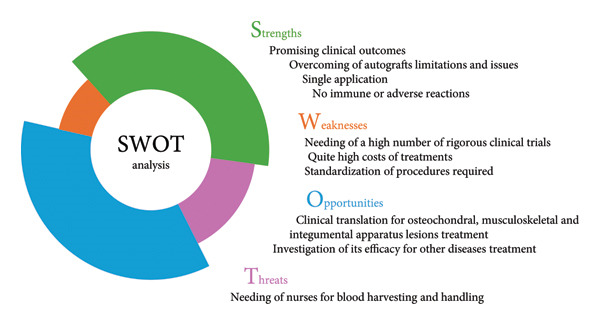
SWOT analysis on blends of PRP/PRF and collagen.

The efficacy of blends of collagen and platelet concentrates in the treatment of several diseases started to be evaluated only in recent years. Indeed, no clinical studies were performed before 2009. The increasing number of in vitro and preclinical studies suggested the high interest in this blend and the substantial potential of its outcomes. As with all approaches, PRP/Coll blends own strengths, opportunities, as well as weaknesses and threats (Figure 5). Although pilot, concluded clinical studies highlighted the effectiveness of such strategies in the treatment of some diseases and the advantages associated with their use. In particular, based on the studies included in this systematic review, this approach seems to be a promising alternative for the treatment of gingival recessions (e.g., periodontitis‐related defects), musculoskeletal lesions (e.g., rotator cuff tears, tennis elbow), and nonhealing wounds (i.e., pressure sores, diabetic foot ulcers). PRP/Coll blends were found to be less promising for bone regeneration. However, further and higher‐quality clinical trials are needed to confirm the preliminary findings and establish robust evidence regarding the efficacy and safety of the interventions. All conducted studies suffered from several limitations (e.g., small number of enrolled participants, lack of an adequate control group, short follow‐up period, few details on the blends preparation) which do not allow us to assert with certainty the real superiority of this approach compared to conventional therapies. Conducting well‐designed randomized controlled trials with adequate statistical power, standardized outcome measures, and long‐term follow‐up is essential to draw definitive conclusions and support clinical recommendations.

A further limitation concerns the lack of standardization in PRP/PRF preparation and reporting. All clinical studies followed different methods (in terms of devices, anticoagulants, blood volumes, and centrifugation protocols), producing concentrates that are labeled similarly but vary markedly in platelet and leukocyte content. Such variability directly affects growth factor release and inflammatory activity, making cross‐study comparisons difficult and limiting the strength of clinical conclusions on PRP/Coll and PRF/Coll blends. Lastly, another limitation derives from collagen types, in terms of extraction source and of native structure preservation degree, two aspects that deeply influence collagen bioactivity.

To the best of our knowledge, while PRP/PRF blends with other materials (e.g., hyaluronic acid) are actually under clinical evaluation for osteoarthritis treatment (NCT01697423) [75] or for antiaging (NCT02832583), no clinical studies are available for PRP/Coll or PRF/Coll blends for these applications. The limited amount of data and the lack of evidence for some applications suggested the unexplored potential of this blend and the need of in‐depth investigative analysis. However, a comparative study between the efficacy of microneedling with collagen and with PRP was recently done, showing a significant better clinical and histological wrinkles improvement in patients treated with collagen compared to patients treated with PRP [76]. Nevertheless, the results of this study did not allow us to discard the combination of PRP with collagen, since their combination could significantly enhance collagen activity. Indeed, rigorous clinical studies should be performed on the use of this new blend on wrinkles treatment.

As previously argued, the use of PRP/Coll or PRF/Coll blends as an alternative to conventional therapies could allow us to overcome some of their issues. In particular, the combinatory effect of the blend could overtake the necessity to perform autografts and all related requirements (e.g., donor site availability, graft size, comorbidities restrictions, surgeon expertise, postoperative discomforts, hospitalization time, specialized personnel). However, even though donor site access for autografts would not be any more necessary, it is important to not forget that the collection of blood from patients requires the presence of specialized medical equipment (e.g., nurse to collect blood, trained personnel for platelet concentrates isolation). Although it is a fast procedure that does not require so much time (about 30–60 min), it even adds additional time to the surgical application of ready‐to‐use xenografts. Nevertheless, it should be taken into account that the loss of time required for the preparation and application of the blend is cost‐effective since, unlike conventional therapies, no additional procedures should be repeated during time. As emerged from clinical studies, single treatments of diseases with PRP/Coll and PRF/Coll blends were not only effective but also decreases infection rates and patients’ stress. Consequently, PRP/Coll and PRF/Coll treatments significantly increase patients’ well‐being, beyond decrease treatments costs by reducing outpatient clinic visits that involve disposables, instruments, time as well as qualified personnel.

However, because of the recent investigation on PRP/Coll treatment’s efficacy and the lack of data, surgical procedures on their combination and application are still not standardized. Due to this, these combinatory therapies are quite expensive. Thus, although effective or promising, approaches based on the use of blood derivatives and collagen may not be accessible to all patients, a factor that could create a sociological hedge barrier [47]. The execution of clinical trials is the only way to accurately evaluate the effect of these materials, standardize treatments, and therefore assess their real potential in improving human quality of life.

## 8. Conclusion

Blends of collagen and platelets concentrates demonstrated potential across a wide range of regenerative medicine applications. Their use could circumvent limitations of autografts and reduce postoperative complications. However, the overall quality of evidence remains low due to methodological limitations. This systematic review highlights the emerging role of PRP/Coll and PRF/Coll blends in regenerative medicine. Early clinical results are encouraging, particularly in dental and musculoskeletal fields. However, high‐quality clinical studies are required to confirm their benefit over standard treatments.

## Conflicts of Interest

The authors declare no conflicts of interest.

## Author Contributions

Conceptualization: Luca Salvatore, Domenico Rocco, and Cosimo Saponaro. Data curation: Nunzia Gallo and Domenico Rocco. Formal analysis: Nunzia Gallo, Chiara Kodra, Cosimo Saponaro, and Domenico Rocco. Funding acquisition: Luca Salvatore and Cosimo Saponaro. Investigation: Nunzia Gallo, Cosimo Saponaro, and Alessandro Sannino. Methodology: Chiara Kodra and Cosimo Saponaro. Project administration: Luca Salvatore and Alessandro Sannino. Resources: Cosimo Saponaro and Alessandro Sannino. Software: Nunzia Gallo. Supervision: Luca Salvatore and Domenico Rocco. Validation: Luca Salvatore. Visualization: Nunzia Gallo, Luca Salvatore, and Alessandro Sannino. Writing–original draft: Nunzia Gallo, and Chiara Kodra. Writing–review and editing: Nunzia Gallo and Luca Salvatore.

## Funding

This work received no external funding.

## Supporting Information

Table S1. The complete list of all publications that met the eligibility criteria of the study and were included in the final qualitative synthesis. Publication year, issue and PRP/PRF type were indicated, as well as the relative reference number.

## Supporting information


**Supporting Information** Additional supporting information can be found online in the Supporting Information section.

## Data Availability

Data sharing is not applicable to this article as no new data were created or analyzed in this study.
