# Supplementary material for: Regenerative Medicine Advancements: A Systematic Review on the Combinatory Effect of Platelet‐Rich Plasma/Fibrin and Collagen
Source: Int J Biomater. 2026 Jan 12;2026:1679626. doi: 10.1155/ijbm/1679626 (PMC12794683; doi:10.1155/ijbm/1679626)
Supplement: Supplementary file 1 — Supporting Information Additional supporting information can be found online in the Supporting Information section. [file IJBM-2026-1679626-s001.docx]

**Supporting materials**

Table S1. The complete list of all publications that met the eligibility criteria of the study and were included in the final qualitative synthesis.

| **Publication number** |  | **PRP/PRF** | **Year** | **Issue** | **DOI** |
| --- | --- | --- | --- | --- | --- |
| 1 |  | PRP | 2009 | Gingival recession | [52] |
| 2 |  | PRP | 2011 | Periodontitis | [53] |
| 3 |  | PRP | 2012 | Ankylosis | [38] |
| 4 |  | PRP | 2013 | Maxillary sinus lift | [54] |
| 5 |  | PRP | 2014 | Chronic ulcer | [57] |
| 6 |  | PRP | 2017 | Pressure sores | [39] |
| 7 |  | PRF | 2018 | Periodontitis | [59] |
| 8 |  | PRF | 2018 | Fistula | [60] |
| 9 |  | PRP | 2019 | Epicondylitis | [16] |
| 10 |  | PRP | 2021 | Epicondylitis | [42] |
| 11 |  | PRP | 2021 | Ligament reconstruction | [55] |
| 12 |  | PRP | 2021 | Diabetic foot ulcer | [43] |
| 13 |  | PRF | 2021 | Gingiva insufficiency | [61] |
| 14 |  | PRF | 2022 | Gingival recession | [44] |
| 15 |  | PRP | 2022 | Tendinopathy | [15] |
| 16 |  | PRF | 2023 | Tendinopathy | [47] |
| 17 |  | PRF | 2023 | Periapical bone defects | [46] |
| 18 |  | PRF | 2023 | Gingival recession | [48] |
| 19 |  | PRF | 2023 | Maxillary sinus restoration | [62] |
| 20 |  | PRF | 2023 | Gingival recession | [45] |
| 21 |  | PRF | 2023 | Gingival recession | [49] |
| 22 |  | PRF | 2023 | Periodontal intrabony defects | [63] |
| 23 |  | PRF | 2023 | Periodontal intrabony defects | [64] |
| 24 |  | PRF | 2025 | Radicular cyst | [65] |
| 25 |  | PRP | 2025 | Peripheral nerve injury | [56] |
| 26 |  | PRP | 2025 | Neuropathic pain | [58] |
